# National Life Expectancy Lags Behind Benchmark Progress and the Role of Smoking: An International Comparison

**DOI:** 10.1007/s10680-025-09760-8

**Published:** 2025-12-09

**Authors:** Marcus Ebeling, Frederik Peters

**Affiliations:** 1 Max Planck Institute for Demographic Research, Rostock, Germany; 2 Max Planck–University of Helsinki Center for Social Inequalities in Population Health, Rostock, Germany, and Helsinki, Finland, Germany; 3 Karolinska Institute, Stockholm, Sweden; 4 Hamburg Cancer Registry, Hamburg, Germany

**Keywords:** Smoking, Life expectancy, Time lag, Low-mortality countries, Longevity Frontier

## Abstract

Countries with low mortality show uneven success in improving longevity. Smoking—still a dominant health risk—is a major determinant of these divergent trajectories. Our study aims to determine: a) the continuing influence of smoking on national mortality trends, and b) the extent to which other factors are preventing countries from realizing their health potential. Using mortality data from 20 low-mortality countries (1950–2019), we quantify life expectancy and age-specific mortality differences as calendar years behind the current longevity frontier, defined as record smoking-eliminated life expectancy. We find that current life expectancy largely reflects smoking-eliminated records from two decades ago, with a notable gender paradox across most countries: men are moving closer to the optimal health benchmarks, while women are drifting further away, although men still bear a greater burden of past smoking. While longevity leaders differ from laggards mainly in mortality at advanced ages, laggards show also extensive developmental delays throughout most of the working ages and the second half of life. Our results highlight the diminishing effect of smoking and the role of additional adverse factors in delayed mortality improvement. However, the positive effects from smoking declines have not yet been fully realized, as evidenced by the still comparatively high reduction in development delays after eliminating smoking. Nevertheless, the realization of further health potential largely depends on countries’ ability to manage health in old age, including the increasing burden of chronic disease.

**Supplementary Information:**

The online version contains supplementary material available at 10.1007/s10680-025-09760-8.

## Introduction

A major driver of delayed mortality improvements across developed countries was and continues to be the tobacco epidemic. Tobacco smoking accounted for 175 million deaths from 1990 to 2021 worldwide and is deemed to be the single most important health risk in low-mortality countries and still the leading cause of premature mortality (Bryazka et al., 2024; Mons & Kahnert, 2017; National Center for Chronic Disease Prevention and Health Promotion (US) Office on Smoking and Health 2014). The impact of the tobacco epidemic has shown to be a major underlying factor of irregularities in mortality change during the past century up until today (Janssen et al., 2021a, 2021b; Peters et al., 2016). The epidemic affected countries at different points in time with earlier smoking uptake in men, and with a lag of several decades, in women (Thun et al., 2012). The devasting effects of smoking are widely acknowledged and anti-smoking campaigns are in place in most countries worldwide, which led to a general decrease in the prevalence of smokers (Nogueira et al., 2018; Reitsma et al., 2017).

However, the design and scope of anti-smoking campaigns vary widely, and as a result smoking prevalence is declining at different rates across countries (Dowd et al., 2025). Yet, regardless of current smoking habits and policies, the detrimental effects of past smoking are long-lasting and must be considered when assessing country-specific mortality trends (Janssen et al., 2013). Thus, even if the number of smokers is currently declining, the past smoking history of a population still influences its changes in mortality (Preston & Wang, 2006).

The complex effects of smoking on mortality create a dynamic in which a country’s current life expectancy depends not only on the timing of smoking policies, but also on its historical smoking trajectory. It has been argued that declining smoking rates and resulting mortality improvements have been a key driver of life expectancy gains in recent decades. However, as this potential has been increasingly realized, the recent slowdown in mortality improvements observed in many countries may reflect diminishing returns from smoking declines (Janssen et al., 2021a, 2021b; Lopez & Adair, 2019; Mehta et al., 2020). Therefore, smoking remains one of the main reasons why some countries lag behind the longevity frontier, while others become longevity leaders over time. In other words, smoking remains one of the main reasons why countries are not able to reach their full health potential. At the same time, we can only properly evaluate other factors that prevent populations from reaching their full health potential by quantifying and removing smoking’s distorting impact on mortality.

We define the health potential of a population as the maximum health status that a population could achieve under ideal conditions with optimized social determinants of health, such as education, access to healthcare, and lifestyle. The concept of a population’s health potential is closely related to the longevity frontier. The longevity frontier is often considered the maximum achievable life expectancy under current conditions. It has previously been measured by record life expectancy, which is defined as the highest observed life expectancy in a given year (Oeppen & Vaupel, 2002; Vaupel et al., 2021). In this study, we use a similar concept to define a progress benchmark against which we compare country-specific values, namely record smoking-eliminated life expectancy.

The frontier of longevity has been steadily increasing from 46 years in the late nineteenth century to 87 years today (Oeppen & Vaupel, 2002; Vaupel et al., 2021). The pace towards longer life expectancy varied greatly between developed countries with both periods of rapid increase but also slower improvement and also periods with short fallbacks (Goldstein & Lee, 2024; Ouellette et al., 2014). Examples of varying changes include the life expectancy stagnation in the Netherlands between the early 1980s and early 2000s (Peters et al., 2015), the life expectancy deterioration among Danish women from the mid-1980s to the mid-1990s (Lindahl-Jacobsen et al., 2016), which are both related to smoking, the underwhelming development of German life expectancy compared to other countries since reunification (Jasilionis et al., 2023), and the recent stagnation in the life expectancy in the US and the UK since 2010 (Mehta et al., 2020; Polizzi et al., 2024; Woolf, 2023). These are only a few examples of developed countries struggling to keep pace with the pioneering populations on their path to higher longevity, and where smoking has been argued to play a role.

National progress toward the longevity frontier reflects not only overall population health trends but also the changing dynamics within constituent subpopulations. For example, socioeconomic differences in mortality are growing in several high-income countries and are projected to widen even more in the future (Long et al., 2023; van Baal et al., 2016). These differentials partly reflect differentials in the level and the trend of smoking, which is known to exhibit a strong social gradient (Östergren, 2022). Yet, the extent to which smoking drives the socio-economic inequalities in life expectancy varies across studies (Hemelrijck et al., 2024; Janssen et al., 2015; van Raalte et al., 2015). This variation in smoking’s impact across socioeconomic groups—and across countries—highlights the importance of understanding smoking’s role in national lags behind the longevity frontier.

Although smoking is not the only factor preventing populations from reaching their health potential, it remains one of the most significant modifiable risk factors. However, several other factors with a negative impact on mortality improvements have gained momentum in the recent past. These factors are often social in nature, such as lifestyle, and affect mortality to varying degrees. Beyond the tobacco epidemic and socioeconomic deprivation, rising levels of obesity are considered to be one of the most important threats to population health (Janssen et al., 2020; Mehta, 2023). Obesity increases the risk of several types of diseases, such as cardiovascular disease (CVD). CVD are a major cause of death and their development is crucial for overall mortality change (Lopez & Adair, 2019). A decreasing impact of smoking on mortality could offset the increasing negative effects of rising obesity. However, the extent to which this is happening is unclear. Moreover, recent developments, such as the worsening health of younger cohorts—also known as ‘generational health drift’—partly driven by rising obesity prevalence, may suggest that the positive effect of declining smoking on mortality may be limited. In addition, the health of these cohorts is being affected by additional health burdens such as mental illness (Dowd et al., 2025). Evidence for the “generational health drift” can be found in several high-income countries, with the US and UK showing the clearest pattern (Gimeno et al., 2025; Jivraj et al., 2020; Zheng & Echave, 2021).

Taken together, the evidence to date suggests that the effect of smoking on mortality change appears to be diminishing and that additional health burdens, such as the obesity epidemic or social deprivation, are gaining ground (Dowd et al., 2025). However, even if the effect is diminishing, smoking remains one of the most important modifiable factors preventing populations from achieving their optimal health potential (Lhachimi et al., 2016). In this study, we therefore define record smoking-eliminated life expectancy as quantitative benchmark of achievable health potential over the past 70 years. Similar to record life expectancy, we define record smoking-eliminated life expectancy as the highest smoking-eliminated life expectancy observed in a given year. With record smoking-eliminated life expectancy, we use a progress benchmark that reflects a counterfactual estimate of the longevity frontier if smoking would have not affected mortality trends in the considered countries. Hence, we are using a longevity benchmark that is free from one of the greatest threats to population health in the twentieth century.

By comparing the observed and smoking-eliminated performance of countries against this benchmark, our study aims to determine: a) the extent to which smoking continues to influence national mortality trends, and b) the extent to which factors other than smoking are preventing countries from reaching their health potential. These questions are particularly timely, as understanding these dynamics could shed light on pathways to closing persistent health gaps and identifying leaders and laggards on the path to the health optimum.

Rather than relying on conventional absolute or relative differences in mortality measures, we approach these questions by analysing mortality differences through time lags between countries and the reference trend. Although lags have been used previously in mortality research, their application offers distinct advantages by complementing the current practice of comparing differences in mortality measure with a temporal forecasting perspective (Stolnitz, 1955). The lag perspective provides a simple, general and intuitive concept to express in calendar years the developmental lag of a population relative to a reference population or trend. Furthermore, as changes in mortality and health over time at the population level are inherently dynamic research questions, a delay-based measure such as the time lag addresses this dynamism more directly than static gap measures. In the context of smoking, a delay-based measure may also be more suitable given the long-lasting effect of the smoking histories of populations on their mortality change over time (Janssen, 2020; Preston & Wang, 2006).

The main empirical objectives of this study are threefold: (I) to quantify how much countries are lagging behind the current frontier of longevity, (II) how much of the lag is attributable to smoking, and (III) where are the future potentials for closing the lag, which we will do by quantifying the lag of differences in age-specific mortality with and without controlling for the effect of smoking.

## Methods

### Data

Age, sex- and country-specific death counts and person-years of exposure were extracted from the Human Mortality Database (HMD) for the years 1950 until 2019 (University of California, Berkeley (USA), and Max Planck Institute for Demographic Research (Germany) 2024). Age, sex- and country-specific lung cancer deaths between 1950 and 2019 were obtained from the WHO Mortality Database (World Heath Organization (WHO) 2024). The codes used to identify lung cancer in the WHO data according to the different versions of the International Classification of Diseases and Related Health Problems (ICD) were “162, 163” for ICD-7, “162” for ICD-8, “162” for ICD-9, “C33” and “C34” for ICD-10 (Janssen & Kunst, 2004).

Deaths and exposures have been grouped in five-year age groups (0, 1–4, 5–9, …,80–84, 85 +). We included only countries with sufficient information from both sources of data up until 2019 to focus on secular trends unaffected by the COVID-19 pandemic. This resulted in a list of 20 countries in the analysis (with ISO3 country codes: AUS—Australia, AUT—Austria, BEL—Belgium, CAN—Canada, CHE—Switzerland, DEU—Germany, DNK—Denmark, ESP—Spain, FIN—Finland, FRA—France, GBR—United Kingdom, ITA—Italy, IRL—Ireland, JPN—Japan, NLD—Netherlands, NOR—Norway, NZL—New Zealand, PRT—Portugal, SWE—Sweden, USA). Lung cancer death rates were computed by dividing lung-cancer deaths by exposures and smoothed over age and time using a Poisson regression approach. The two-dimensional thin-plate splines over age and year were applied to account for calendar years with missing data for lung cancer and to reduce random fluctuations in smaller countries (Wood, 2003).

### Estimating Smoking-Eliminated Mortality and Life Expectancy

The estimated smoking-eliminated life expectancy is based on death rates from which all smoking-related deaths have been removed. For computing smoking-eliminated death rates, the age-specific attributable fraction of deaths due to smoking must be removed from the observed age-specific deaths. To estimate the attributable fraction, we applied the coefficients from the modified Preston-Glei-Wilmoth approach suggested by Rostron (Preston et al., 2010; Rostron, 2010). This approach includes an age-period interaction. We also applied an extension by Martikainen et al. to extrapolated the coefficients to more age groups (Martikainen et al., 2014). We assumed the attributable fraction at ages below 45 to be zero.

The Preston-Glei-Wilmoth approach is, at its core, a regression-based model in which deaths from causes other than lung cancer are modelled as a function of lung cancer deaths, taking into account country, sex, age, and period. Based on the coefficients from this model, the smoking-attributable fraction of deaths from causes other than lung cancer is calculated for countries, calendar years, and separately for women and men. Additionally, the smoking-attributable fraction of lung cancer deaths is computed by comparing observed lung cancer death rates with expected rates for non-smokers, based on data from the American Cancer Society Cancer Prevention Study II (Calle et al., 2002). A more detailed description and evaluation of the estimation of smoking-eliminated death rates is presented elsewhere (Rostron, 2010). Note, the approach of Preston-Glei-Wilmoth lead to virtually similar fractions than a fundamentally different method developed much earlier by Peto et al. (1992). Life expectancy and smoking-eliminated life expectancy were computed using standard lifetable methods for abridged lifetables using all-cause death rates and smoking-eliminated death rates as input (Preston et al., 2001).

### Estimating Mortality Differences Based on Time Lags

**Fig. 1:**
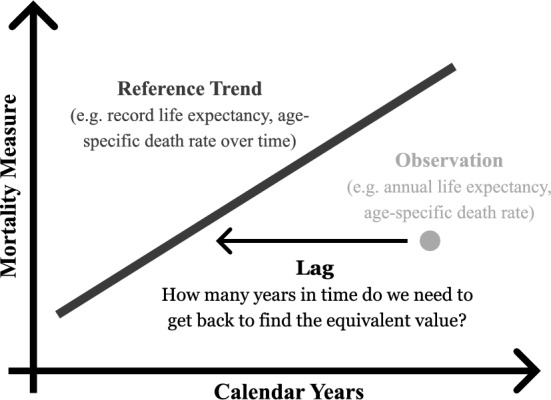
Illustration of the Lag Calculation

We use time lags to assess progress in survival (see Fig. 1). The idea was proposed by Stolnitz more than 60 years ago (Stolnitz, 1955). He used this approach to study the lags in mortality decline in non-Western countries relative to Western countries. Surprisingly, we are not aware of any other study using this approach since then. More recently, Goldstein and Wachter have used a similar concept to study the relationship between cohort and period life expectancy within countries (Goldstein & Wachter, 2006). They defined the lag as “how far back in time from the current period we have to go to find a cohort with equivalent life expectancy” [Goldstein & Wachter, 2006, 18, p. 259]. The general idea of time lags has also been used to assess the decline in age-specific mortality over human history by expressing the improvement in ages with equivalent levels of mortality, usually called ‘equivalent ages’ (Burger et al., 2012). While these earlier studies focused on detailed aspects of mortality and survival, we will use time lags for a cross-country comparison of overall survival progress.

As shown in Fig. 1, and similar to previous work, we define the lag as “how many years back in time do we have to go to find the equivalent value [in life expectancy or death rates]”. We use smoking-eliminated life expectancy—the highest observed smoking-eliminated life expectancy in a given years across our set of countries—as the reference trend and benchmark progress. In a second step, we use also the time series of age-specific death rates in the annual “record” lifetables to estimate the lag in death rates. The lag estimation is performed between the country-specific observed and smoking-eliminated life expectancy and death rates and the respective reference trends.

In order to identify a unique lag, the reference trend must fulfil certain requirements. To rule out the possibility of more than one equivalent value, the reference time series must change monotonically over time. However, this could be seen as a minor problem, as we see fairly steady improvements in most measures of the longevity frontier, such as life expectancy. However, a constant change over time is not necessary to calculate the lag because the empirical values of the reference and comparison populations can be compared directly. For instance, once estimated, a lag of 15 years can be interpreted as follows: “Current life expectancy in Country Y corresponds to levels that the reference trend reached 15 years ago.” Higher lags indicate greater delays in mortality development and refer to countries with lower life expectancy or higher mortality rates than the reference trend.

### Analytical Approach

Our analytical approach consists of four main steps. *First*, we estimate record life expectancy for both observed and smoking-eliminated death rates for women and men to identify and compare both trends. *Second*, we calculate the separate lags for observed and smoking-eliminated country-specific life expectancy in 2019 and the respective reference trend. We also estimated the lag for the average death rates across countries. Sex-specific record smoking-eliminated life expectancy will serve as our reference trend in the lag estimation and thus, as our measure of the current global maximum health potential of populations. This comparison reveals the general lag of populations with and without smoking elimination. Record smoking-eliminated life expectancy can be interpreted as an estimated counterfactual of record life expectancy in which the populations under consideration would have no smoking history. This approach rests on a concept similar to estimating excess mortality, where excess mortality is defined as the deviation of observed mortality from a baseline reflecting mortality in the absence of an external stressor, such as the Covid-19 pandemic (Nepomuceno et al., 2022). Thus, by subtracting the lag in observed life expectancy from the lag of smoking-eliminated life expectancy, we can get an estimate of the effect of smoking on the current lag in country-specific mortality. We estimate the lags using the approx() function in R version 4.2.3. (R Core Team, 2023), which has the advantage of incorporating the empirical trajectory, where periods of faster and slower increases in the reference trend are considered in the lag estimation. *Third*, we repeat this analysis for country-specific observed and smoking-eliminated life expectancy in 2000 and compare the change in the respective lags to understand country-specific changes over time. *Fourth*, we perform lag estimation for each age-specific death rate separately. This analysis will shed light on how age-specific mortality contributes to the overall lags in life expectancy and for death rates above 45, how lags in age-specific mortality change when smoking is eliminated. This is done for each sex separately, using the time series of smoking-eliminated age-specific death rates from the annual record lifetables in smoking-eliminated life expectancy. Thus, death rates above the age of 45 in the reference trend are smoking-eliminated death rates. To ensure robust lag estimation with our available data, the death rates across the record holding populations were smoothed using a quasibinomial() generalized additive model with years as covariates. The lag was estimated for both observed and smoking-eliminated death rates. As a supplemental analysis and to understand changes over time, the lag for 2000 is estimated for each death rate in that year and compared with the observed lag for the death rate in 2019 (results in the supplemental materials). Data and code are openly available in a public repository (10.17605/OSF.IO/EQKS2).

## Results

### Record Life Expectancy

**Fig. 2:**
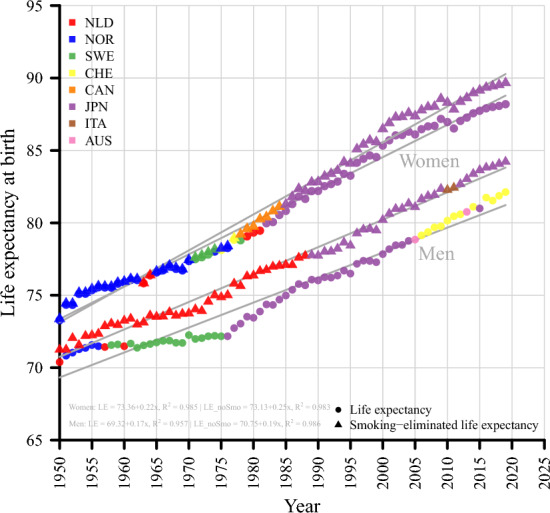
Record observed and smoking-eliminated life expectancy, women & men, Years 1950–2019

Figure 2 shows record observed and smoking-eliminated life expectancy for women and men, respectively. Dots are the respective observed value, while triangles depict the smoking-eliminated value. We also fitted a simple linear regression to highlight the general trend. R^2^ values in all four models are higher than 0.95. Overall, eight different countries contributed to the record trends over the last 70 years.

Both record observed and smoking-eliminated life expectancy increased between 1950 and 2019 for both men and women. From an initial level of 73 years (women) and 70 years (men) in 1950, record observed life expectancy increased to 88 (women) and 82 (men) years in 2019, while the smoking-eliminated are 73 in 1950 and almost 90 in 2019 for women as well as 71 in 1950 and 84 in 2019 for men. Based on an estimation via linear regression (lower left corner), this corresponds to an annual increase of 2.2 years per decade (observed) and 2.5 years per decade (smoking-eliminated) for women and 1.7 years per decade (observed) and 1.9 years per decade (smoking-eliminated) for men. However, Fig. 1 also illustrates that the pace of increase varied over time.

Since the mid-1980s, Japanese women have been the record holders among women for both types of life expectancy, while Japanese men only appear as record holders after eliminating the effect of smoking. For observed life expectancy, Swiss men have shown the highest values over the last 15 years. Comparing the difference between observed and smoking-eliminated values, it is clear that smoking had a much stronger effect on the benchmark progress of men than of women. However, the difference between record smoking-eliminated and observed life expectancy for women seems to have increased in recent years.

### Smoking-Eliminated and Observed Time Lags in 2019

**Fig. 3:**
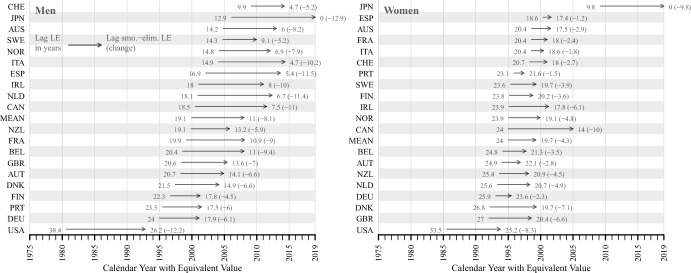
Lag and equivalent year (horizontal axis) of country-specific observed and smoking-eliminated life expectancy in 2019 as compared to record smoking-eliminated life expectancy (1950–2019), women & men. *Note:* Countries are ordered based on the lag in observed life expectancy in 2019. MEAN refers to the lags of observed and smoking-eliminated life expectancy, which are calculated based on the average age-specific death rates across all countries

Figure 3 shows the time lag between the observed and smoking-eliminated life expectancy by country and sex in 2019 and record smoking-eliminated life expectancy (reference trend). The horizontal axis shows the calendar year in which the equivalent life expectancy value in the reference trend was found. The numbers at the beginning of the arrow (observed) and at the end (smoking-eliminated) represent the time lag in calendar years as well as the difference between the observed and smoking-eliminated life expectancy lags (in brackets). Countries are ordered based on the lag in observed life expectancy.

In the case of Swiss, for instance, the numbers can be interpreted as: observed life expectancy in 2019 corresponds to the level of record smoking-eliminated life expectancy around 9.9 years ago. Their observed life expectancy in 2019 thus corresponds to level of the reference trend in 2009 (horizontal axis). After eliminating smoking, the lag decreased to 4.7 years, a decrease of 5.2 years.

The reduction in the time lag after eliminating smoking is greater for men than for women. Despite the apparently smaller effect of smoking, time lags are generally longer for women than for men. The cross-country variation in the time lag between countries is also smaller for women than for men. For both women and men, the observed life expectancy in the majority of countries in 2019 corresponds to values of record smoking-eliminated life expectancy recorded in the 1990s. Moreover, the differences between the lags of observed and smoking-eliminated life expectancy shows also greater variation among men compared to women.

For both women and men, the time lag is greatest in the US. Observed life expectancy in the US in 2019 corresponds to the value reached by record smoking-eliminated life expectancy almost four decades ago (38.4 years) for men and more than three decades ago (33.5 years) for women. Eliminating smoking reduces the gap to 26.2 years for men and 25.2 years for women. Japanese women and men, who are the record holders in smoking-eliminated life expectancy in 2019, also show a significant reduction in the time lag of about a decade when comparing observed and smoking-eliminated life expectancy. Japanese men are also the record holders only after eliminating smoking, as Swiss men have the highest observed life expectancy in 2019 among the countries considered. Italian men and Canadian women are the two populations closest to the record after elimination of smoking. However, even after adjusting for smoking, Canadian women are still 14 years behind, while Italian men are only five years behind the record benchmark.

### Changes in the Time Lag Between 2000 and 2019

**Fig. 4:**
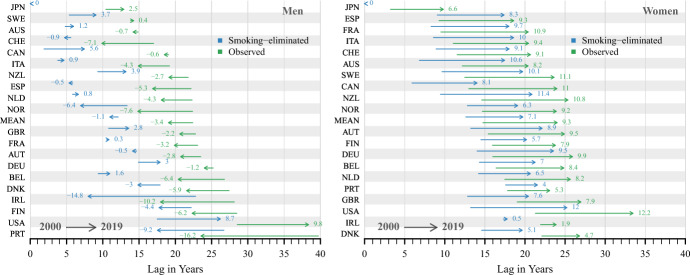
Lag of country-specific observed and smoking-eliminated life expectancy in 2000 and 2019 as compared to record smoking-eliminated life expectancy (1950–2019), women & men. Note: Countries are ordered based on the lag in observed life expectancy in 2000. MEAN refers to the lags of observed and smoking-eliminated life expectancy, which are calculated based on the average age-specific death rates across all countries

Figure 4 shows the change in the time lag between the reference trend (record smoking-eliminated life expectancy) and the country-specific observed (green arrow) and smoking-eliminated (blue arrow) life expectancy in 2000 and 2019. The numbers are the absolute change in the time lag. Countries are ordered based on the lag in observed life expectancy in 2000.

Figure 4 shows very different trends for men and women. For women, the observed lag changes show that all analysed countries are experiencing increasing delays and have increasing lags over time when compared with the record smoking-eliminated life expectancy. Irish women are an exception and are the only female population to improve at almost the same pace as the reference, as shown by the small changes in the time lag. US women again show an exceptional pattern and experienced the largest increase in the lag of 12 years between 2000 and 2019. For women in most countries, increases in the lag of observed life expectancy exceed those of smoking-eliminated life expectancy. Most countries show increases in the observed life expectancy gap of between nine and 11 years, and slightly smaller increases in the smoking-eliminated life expectancy lag. This suggests a relatively uniform pace of improvement across the comparison countries, but a faster increase in record smoking-eliminated life expectancy for women, which underpins the exceptional development of Japanese women in the last decades.

Women in countries with the smallest lags for both measures of life expectancy in 2000 show the greatest increase in the gap. For example, the smoking-eliminated life expectancy of Italian women had an initial gap of 9 years in 2000, which increased by 10 years to a gap of 19 years in 2019, while the gap in observed life expectancy increased by 9.4 years to 21 years in 2019. Dutch women, however, only experienced an increase of 6.5 years (smoking-eliminated) and 8.2 years (observed) from initial lags of around 15 years in 2000. It is noteworthy that female populations with a comparatively higher lag in smoking-eliminated life expectancy in 2000 showed smaller increases in the lag compared with countries with a smaller initial lag.

The pattern for men is very different from that for women. With the exception of US men, almost all countries show either stagnating or decreasing lags between 2000 and 2019. Many countries also show different trends in the lag between observed and smoking-eliminated life expectancy. For most countries, the observed life expectancy lag decreased between 2000 and 2019, while the smoking-eliminated life expectancy lag stagnated or slightly increased. This is different from women, where the change in both lags was in the same direction. Similar to Irish women, Irish men also show one of the most favourable trends, with a decrease of almost 15 years in the smoking-eliminated life expectancy lag to a lag of 8 years in 2019 and a decrease of 10 years in the observed life expectancy to a lag of 18 years in 2019. Portuguese men also show significant reductions in the lags of observed and smoking-eliminated life expectancy.

For men in many countries, including Australia, Switzerland, Italy, Spain and the Netherlands, the lag in smoking-eliminated life expectancy remained almost constant between 2000 and 2019, with a lag of around five years. Men in Sweden, Canada, the United Kingdom and Germany are the only populations that experienced a comparatively smaller but more pronounced increase in the lag in smoking-eliminated life expectancy, with an increasing lag of three to six years. Similar to women, US men show a very different trend from all other countries. For US men, the observed and smoking-eliminated life expectancy lags increased by about nine to ten years between 2000 and 2019.

### Lags in Age-Specific Death Rates

**Fig. 5:**
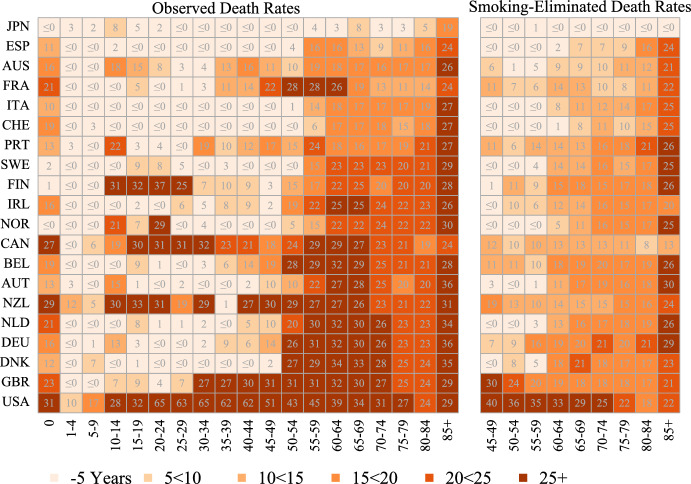
Lag (in years) of country-specific observed and smoking-eliminated age-specific death rates in 2019 as compared to the time series of age-specific death rates from the annual record smoking-eliminated life tables (1950–2019), women. *Note:* Countries are ordered based on the lag in observed life expectancy in 2019. Numbers indicate the specific lag estimate with the open value bins “ ≤ 0” indicating values below or equal to zero, with negative values meaning that a certain death rate is lower compared to the reference death rate in 2019, and the open value bin “69 ≤ ” indicating lags equal or higher to 69 calendar years

**Fig. 6:**
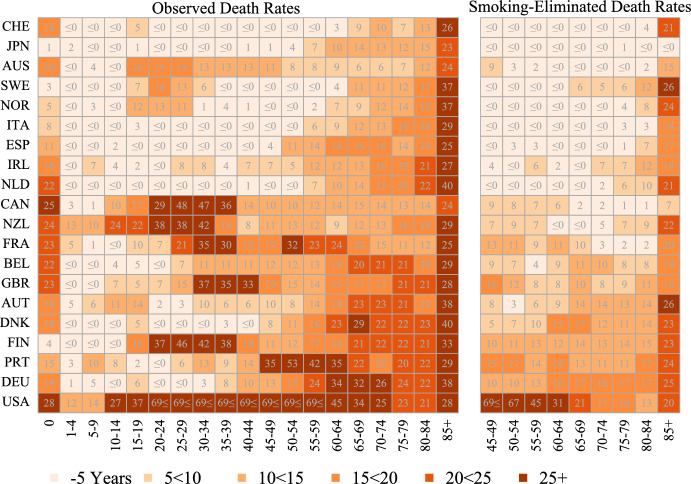
Lag (in years) of country-specific observed and smoking-eliminated age-specific death rates in 2019 as compared to the time series of age-specific death rates from the annual smoking-eliminated record life tables (1950–2019), men. *Note:* Countries are ordered based on the lag in observed life expectancy in 2019. Numbers indicate the specific lag estimate with the open value bins “ ≤ 0” indicating values below or equal to zero, with negative values meaning that a certain death rate is lower compared to the reference death rate in 2019, and the open value bin “69 ≤ ” indicating lags equal or higher to 69 calendar years

Figures 5 and 6 show the lag between country-specific observed and smoking-eliminated age-specific death rates in 2019 and the respective fitted time trend of age-specific death rates from the “record” smoking-eliminated lifetables. Darker read colors indicate higher lags. Numbers indicate the specific lag estimate with the open value bins “ ≤ 0” indicating values below or equal to zero, with negative values meaning that a certain death rate is lower compared to the reference death rate in 2019, and the open value bin “69 ≤ ” indicating lags equal or higher to 69 calendar years. Countries are order based on their lag of observed life expectancy in 2019 (similar to Fig. 3).

Women in countries with a small lag in observed life expectancy show almost no differences in death rates for children, adolescents and adults. However, in these countries, which include, for example, Spain, Australia or Italy, death rates at age 55 + are 15 to 20 years behind the reference trend. These countries also show marked lags of around 25 years in the death rates at age 85 + . The age-specific lags for French women show a slightly different pattern compared to the other vanguard populations, with particularly pronounced lags at ages 45–64, but at the same time French women also have the smallest lags among the leading countries for death rates at ages 70–84. With the exception of France, which shows stronger reductions, the lag of smoking-eliminated death rates is only slightly lower than the lag of observed death rates.

Women in countries with higher lags in observed life expectancy (e.g. Canada and below) show particularly high lags in death rates at ages 50 +. For most countries, these lags are much smaller when smoking-eliminated death rates are considered. Women in the United States and the United Kingdom show an exceptional pattern with particularly high lags at working ages, including ages below 50. The analysis also reveals country-specific age characteristics. For example, French and Belgian women aged 50–64 have much higher lags than younger and older women. Similarly, Finnish and Canadian women between the ages of 10 and 29 show increased lags. In all countries, mortality at the open age of 85 + is particularly lagging behind the reference values. The lap for this age group was only slightly reduced for smoking-eliminated death rates.

Men in the best performing countries also show very low lags at most ages below 60 and higher lags thereafter. The exception is Australian men, who have high lags at working ages but low lags at ages 60 and over. For the best performing countries, almost all lags in observed death rates disappear for smoking-eliminated death rates. For countries at the lower end of the ranking (e.g. Canada and below), large lags are already observed at ages below 60. These lags are considerably reduced when smoking-eliminated death rates are considered, but they remain high, with most age-specific smoking-eliminated death rates lagging 10 to 15 years behind the level of the respective reference trend. US men are again an extreme outlier in the comparison, with working-age death rates similar to levels of the reference trend more than 69 years ago. For all countries, the longest lags are observed for death rates at age 85 + , even after eliminating smoking. We also find high variation in the lags of infant mortality across countries and for both women and men.

Changes in the lag of observed and smoking-eliminated age-specific death rates between death rates in 2000 and death rates in 2019 are shown in Supplementary Figs. 1 and 2. For males, the change shows a reduction in the lag of observed death rates at almost all ages. The reduction is particularly strong for the 15–60 age group, while the lags for death rates at higher ages have decreased less or even stagnated, as in the case of the death rate in the open age group 85 + . For smoking-eliminated death rates, the pattern is more mixed. For women, the reduction in the observed death rates below the age of 60 is much less pronounced and is mostly present below the age of 50, while the death rates at higher ages show a general increase in lags. The same applies to smoking-eliminated death rates.

## Discussion

Smoking has been, and continues to be, one of the major threats preventing populations from achieving their health potential. In this study, we examined the extent to which smoking continues to influence national mortality trends and the extent to which factors other than smoking are preventing countries from reaching their health potential. We did this by comparing observed and smoking-eliminated life expectancy and age-specific death rates in 20 countries with record smoking-eliminated life expectancy and age-specific death rates over the past 70 years as a quantitative measure of achievable health potential.

Our analysis revealed four main findings. *First*, record smoking-eliminated life expectancy increased slightly faster than record observed life expectancy. In addition, the differences between record observed and record smoking-eliminated life expectancy were greater for men than for women. Japanese women have held the record for both types of life expectancy since the 1980s. Japanese men have held the record only for smoking-eliminated life expectancy, while Swiss men show the highest observed values. *Second,* we found a larger difference in the effect of smoking on current life expectancy for men than for women. The lags in observed life expectancy—which are generally lower for men—show a much larger decline after removing smoking-attributable mortality than for women. For women, current values of both smoking-eliminated and observed life expectancy are almost two decades or more behind our reference trend. For men, the lags are somewhat smaller, especially after eliminating the effect of smoking. *Third,* the lag in observed and smoking-eliminated life expectancy for men stagnated or even decreased between 2000 and 2019 compared to the reference trend, while the lag for women increased significantly between 2000 and 2019. The changes in the lag suggest that men in the comparison countries are improving at a similar or even faster pace than the reference trend, while women are improving at a slower pace. For women, this particularly highlights the exceptional nature of the trajectory of Japanese women. *Fourth*, in order to be a leader in life expectancy and move closer to the benchmark progress, it is necessary to eliminate almost all delays in improvement at ages below 60. The greatest potential for future reductions in developmental delays is concentrated at the age of 85 and over. The countries with the greatest overall delays also show significant delays at several other ages, even after adjusting for the effect of smoking. This points to the importance of adverse factors beyond smoking for the underperformance of these countries, which are mostly rooted in the working-age population, like in the case of the US or the UK (National Academies of Sciences, Engineering, and Medicine 2021; Timonin et al., 2024).

Smoking had a much stronger effect on the performance of record life expectancy for men than for women. For men, the difference between the smoking-eliminated and observed record life expectancy is evident for almost the entire observation period. For women, both began to diverge in the early 1980s, but at a very slow pace up until today. This difference highlights the varying timing of the smoking epidemic for men and women, which previous studies estimated to be almost 25 years apart from each other (Janssen, 2020). The results also show that smoking has a lasting impact on national mortality levels. For instance, a previous study found evidence that, in an analysis across several countries, a lag of 25 years between smoking prevalence and subsequent mortality was statistically most plausible (Pampel, 2005). The result also shows the reach of smoking, that even Japan—the longevity vanguard—show substantial and lasting mortality distortions by smoking (Funatogawa et al., 2013). For men, the almost parallel change in record smoking-eliminated life expectancy and record observed life expectancy suggests that at least at the frontier the peak mortality impact has already been reached, since the gap is not growing anymore. A previous study estimated that the smoking-attributable fraction of mortality for men peaked at a level of 33% (ages 35–99) for men, while the fraction was still increasing for women in many countries (Janssen, 2020). Thus, for women, the situation is different, and the peak does not seem to have been reached yet, as also the differences between the two types of record life expectancies seem to be growing.

Current life expectancy in most of the countries compared is similar to the smoking-eliminated record levels of the 1990s and early 2000s. Around these and the following years, record smoking-eliminated life expectancy has continued to increase almost linearly. This shows the potential of countries for further gains in life expectancy, but given the substantial lags after eliminating smoking, it also suggests that likely further modifiable factors may prevent countries from not reaching their health potential (Dowd et al., 2025; Polizzi et al., 2024). Smoking is thus only part of the explanation, and further factors may be different for men and women. The persistence of developmental delays and the different magnitudes for men and women suggests this. Moreover, we did not find a clear relationship between the size of the observed life expectancy lag and the reduction after adjusting for smoking. This again emphasizes the multifaceted context and factors beyond smoking that prevent countries from moving closer to their population health potential.

Our study reveals a striking disparity between men and women in the temporal development of proximity to the longevity frontier. Men got closer to their heath potential, while women moved away from it in the last 20 years. Smoking is contributing to the convergence of men, and likely also to the divergence of women, although at a lower magnitude. For men in many of the compared countries, the lag of observed life expectancy decreased much stronger than the lag for smoking-eliminated life expectancy over the last twenty years. This suggests vanishing benefits of smoking-related mortality improvements for men, which also previous studies suggested (Janssen et al., 2021a, 2021b). Nevertheless, the still comparably high reduction in the lag after eliminating smoking in the recent years also underlines the fact that the positive effects that can be expected from smoking declines have not yet been fully realized. Women could expect more impact of smoking-related mortality improvements in the future but they don’t seem to play a role yet. In fact, the comparable lag increases in observed and smoking-eliminated life expectancy over the last twenty years, suggest that primarily other factors than smoking causes women to lag behind their longevity frontier.

The two countries in the analysis with the most noteworthy development trajectories are the US and Ireland. However, both for opposite reasons. Our analysis emphasized again the exceptionalism of US mortality trends and that the US mortality deterioration is unprecedented. The remaining quarter-century lag in the US after adjusting for smoking shows the role of various additional adverse factors, such as the drug and opioid crisis or the obesity epidemic, that cause the tremendous development delays in US mortality (Aron & Woolf 2013; Woolf, 2023). On the other hand, mortality of Irish men and women revealed one of the most favourable improvements among our set of compared countries. The remarkable development was also noted by other studies and was mostly driven by old age mortality improvements (“Health in Ireland - Key Trends, 2019; Wilkie & Ho, 2024). Importantly, the Irish case shows that progress at the record pace is possible. In fact, Irish men achieved the largest reduction in lags between 2000 and 2019, with both observed and smoking-eliminated life expectancy improving faster than the record pace. Irish women similarly maintained improvements comparable to the record pace for smoking-eliminated life expectancy. These outcomes are likely a consequence of Ireland’s anti-smoking campaign “Tobacco Free Ireland”, which is considered one of the most comprehensive tobacco control programs (Joossens & Raw, 2014; Tobacco Policy Review Group, 2013). This demonstrates that populations can overcome smoking-related mortality disadvantages through sustained public health efforts.

Our analysis shows that the vanguard countries have effectively eliminated delays in improvement at younger ages. Additional results shown in the supplementary materials suggest that men have been particularly successful in reducing the gap at younger ages over time. In contrast, women have shown smaller reductions at younger ages and an increase in the gap at older ages over time. This elimination at younger ages appears to be crucial because favorable mortality at old age seems to only partially compensate for developmental delays at younger ages. The laggard countries show extensive developmental delays throughout most of the second half of life, which persist even after adjustment for smoking, suggesting that other modifiable factors are causing the developmental delay. The future potential for reducing mortality lags is concentrated mainly in the age group 85 + . Consequently, health care systems and approaches to disease management will play a crucial role in reducing mortality at these advanced ages and bringing countries closer to the health potential, as these are also the ages where disease accumulate (Kuan et al., 2019). Nevertheless, the significant reduction in age-specific lags after adjustment for smoking shows that the decline in smoking-attributable mortality will continue to contribute to mortality improvements, at least in the near future.

Our lag-based approach reveals insights that traditional measures of smoking impact cannot capture. While previous studies have documented absolute or relative differences in smoking-related mortality (Bryazka et al., 2024; Martikainen et al., 2014; Reitsma et al., 2017), these approaches show only current impact without addressing the delayed nature of the effects of smoking on population health. The smoking history of a population leaves lasting imprints on both current mortality levels and mortality change over time (Janssen, 2020; Preston & Wang, 2006). This makes a delay-based perspective essential for understanding the full impact of smoking on mortality.

Our findings demonstrate this temporal dimension. Observed life expectancy of Irish men in 2019, for instance, lagged 18 years behind the record trajectory but this lag was reduced to only 8 years after smoking was eliminated. This reveals the substantial contribution of smoking on their mortality disadvantage. The 10-year difference in both lags however also shows that even if Irish men would maintain improvements at the record pace, they would need another decade to reach their health potential if they would have not smoked, and nearly two decades to catch up to the current record value. This illustrates how smoking has not only shaped past mortality outcomes but also continues to slow future progress. For many countries facing similar development delays, eliminating avoidable risk factors like smoking remains an important public health priority, but it is also essential for accelerating convergence toward their full health potential.

The simplicity of the lag, which compares the estimate of an observation with a reference trend, makes it an attractive measure, but it is also its main drawback. The choice of reference trend is always open to criticism and there is always the question of how well a particular reference reflects a general or more appropriate, achievable trend. In our analysis, we used record smoking-eliminated life expectancy at birth as the reference trend. Record observed life expectancy is a recognised measure of the frontier of longevity, and we only remove one of the main factors distorting national mortality trends over the last 70 years (Oeppen & Vaupel, 2002; Vaupel et al., 2021). It is obvious that removing also other modifiable population health threats, like the consequences of alcohol, obesity, or the lack of exercise may result in a more realistic measure of the frontier of longevity. Nevertheless, the analysis for women in particular shows the exceptional nature of Japanese women, with the exception of Irish women, as none of the countries analysed has been able to keep up with the pace of improvement of the female record holders over the last twenty years. However, life expectancy in other countries, such as Hong Kong and South Korea, for which data have not been available for a sufficiently long time, shows that it is possible to approach or even overtake the current and probably exceptional record holders (Ni et al., 2021).

There are several other limitations to our analysis. We do not consider the years of the Covid-19 pandemic. However, we wanted to compare the most recent but also the least distorted estimates, and for most countries the latest available data are still from a period directly affected by the Covid-19 pandemic. It is clear that future analyses will need to consider not only the Covid-19 years, but also the aftermath of the pandemic, and thus the long-term effects on the health of the population. With the open-ended age group 85 + , we also have a very broad age range at the highest ages. Much of the detail that drives the differences between countries, and where the prospects lie, thus remains hidden. Moreover, our approach estimates the effects of smoking at ages 45 and above through a counterfactual setup using smoking-eliminated mortality as a comparison. While we believe this approach achieves a balance between analytical complexity and parsimony, particularly in the context of a cross-country comparison, it may lack the detail necessary to capture all the nuances of the mortality effects of smoking, especially those resulting from its interaction with other factors and those present at ages below 45. However, studying such nuances would likely require a different data basis, such as individual-level data.

## Conclusion

Our research shows the uneven path to longevity among developed countries, with some consistently leading the way and others lagging behind. We found that smoking remains an important determinant of these divergent trajectories, particularly for men. We found that current life expectancy largely reflects smoking-eliminated records from decades ago, with a notable gender paradox: men are moving closer to current achievable health potential, while women are moving further away. Our study also quantifies country-specific differences in a novel way by expressing the lag as the number of calendar years a country is behind the current frontier of longevity. The use of time lags provides a simple and intuitive, yet dynamic way of expressing the development lag of a given population relative to the development of a reference population or trend. Overall, our study highlights the diminishing but still substantial impact of smoking and the role of additional adverse factors in the delayed improvement in mortality. Approaching the current health potential will largely depend on countries’ ability to manage health in old age, including the increasing burden of chronic diseases.

## Supplementary Information

Below is the link to the electronic supplementary material.

Supplementary file1 (PDF 111 KB)

## Data Availability

Data and code for reproducing results are openly available under 10.17605/OSF.IO/EQKS2.
