# Supplementary material for: National Life Expectancy Lags Behind Benchmark Progress and the Role of Smoking: An International Comparison
Source: Eur J Popul. 2025 Dec 9;42(1):1. doi: 10.1007/s10680-025-09760-8 (PMC12708471; doi:10.1007/s10680-025-09760-8)
Supplement: Supplementary file 1 — Supplementary file1 (PDF 111 KB) [file 10680_2025_9760_MOESM1_ESM.pdf]

Supplemental materials: National Life Expectancy Lags Behind Benchmark Progress and the Role of Smoking: An International Comparison

Ebeling, Peters

June 20, 2025

Change in time lags of age-specific death rates

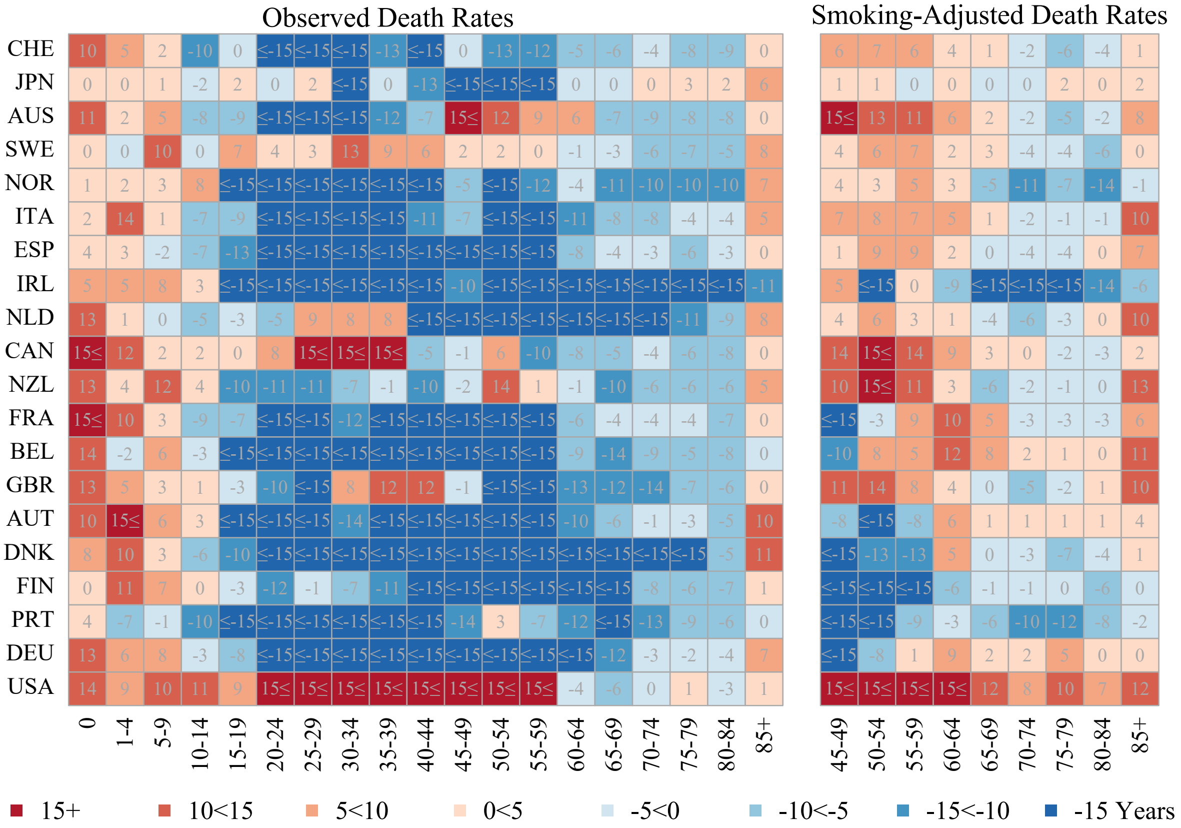

Figure 1: Change in the lag of country-specific observed and smoking-adjusted age-specific death rates between 2000 and 2019 as compared to the time series of age-specific death rates from the annual smoking-adjusted record life tables (1950-2019), men. Note: Countries are ordered based on the lag in observed life expectancy in 2019.

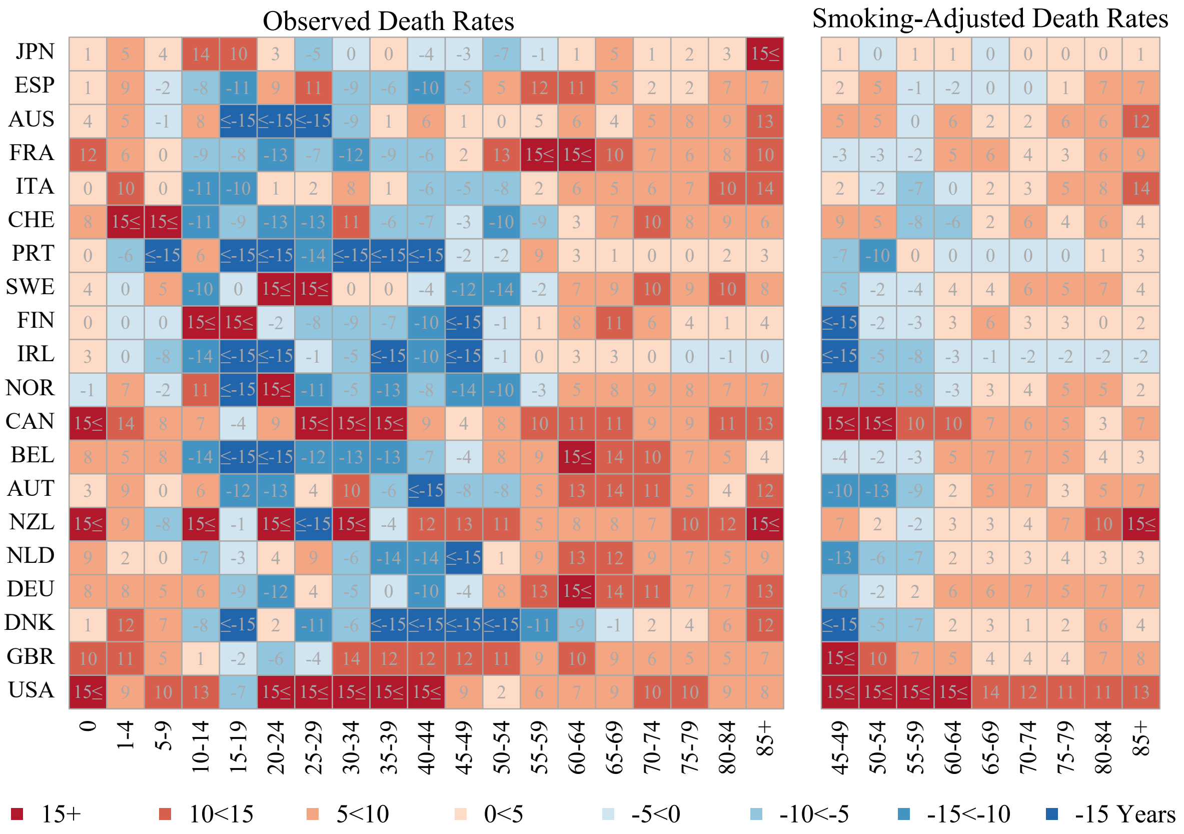

Figure 2: Change in the lag of country-specific observed and smoking-adjusted age-specific death rates between 2000 and 2019 as compared to the time series of age-specific death rates from the annual smoking-adjusted record life tables (1950-2019), women. Note: Countries are ordered based on the lag in observed life expectancy in 2019.
